# Pathological and morphometric characterization of intestinal lesions associated with natural infection by *Oligacanthorhynchus microcephalus* in white-eared opossums (*Didelphis albiventris*)

**DOI:** 10.1016/j.ijppaw.2026.101279

**Published:** 2026-08-25

**Authors:** Julieni Bianchi-Morais, Luis Jhordy Alfaro Quillas, Wuglenya Daislla Martins da Silva, Victoria Luiza de Barros Silva, Dirceu Guilherme de Souza Ramos, Richard de Campos Pacheco, Fernando Henrique Furlan, Nadia Alini Bobbi Antoniassi, e Edson Moleta Colodel

**Affiliations:** a Faculty of Veterinary Medicine (FAVET), Federal University of Mato Grosso (UFMT), Avenida Fernando Corrêa da Costa 2367, Boa Esperança, Cuiabá, Mato Grosso, 78060-900, Brazil; b Laboratory of Veterinary Parasitology and Clinical Analysis, Academic Unit of Agricultural Sciences, Federal University of Jataí, Jataí, Goiás, 75801-615, Brazil; c Animal Pathology Laboratory, Institute of Health Sciences, Federal University of Mato Grosso, Sinop, Mato Grosso, 78550-728, Brazil

**Keywords:** White-eared opossum, Acanthocephaliasis, Granulomatous inflammation, *Oligacanthorhynchus microcephalus*

## Abstract

This retrospective study evaluated 29 free-living white-eared opossums (*Didelphis albiventris*) necropsied between 2022 and 2025. Among 19 adults, 13 (68.4%) presented intestinal remodeling associated with acanthocephalan (*Oligacanthorhynchus microcephalus*) attachment. Parasite burden was determined by counting attached individuals per animal, providing a measure of infection level. Macroscopically, firm nodules (3–11 mm) were observed, mainly in the jejunum, accompanied by luminal dilation and thinning of the intestinal wall. Histologically, the nodules involved mucosa, muscularis, and serosa, containing parasitic sections with necrosis, inflammatory infiltrate, and a thick collagenized capsule (mean 362 μm), with neovascularization; mineralization occurred in 36.4% cases. These findings demonstrate that *O. microcephalus* attachment induces focal chronic granulomatous inflammation and remodeling in *D. albiventris.* To our knowledge, this is the first detailed pathological and morphometric characterization of intestinal lesions caused by this parasite in this host species. Despite the retrospective design and relatively small sample size, the study provides novel insights into the dynamic progression of acanthocephalan-induced intestinal remodeling expanding comparative knowledge of acanthocephalan pathology in neotropical marsupials.

## Highlights

• *Oligacanthorhynchus microcephalus* causes intestinal nodules in *Didelphis albiventris.*
• Parasite attachment induces mural and transmural granulomas
• Lesions evolve through necrosis, inflammation, granulation, and fibrosis
• Fibrous nodules replace intestinal wall layers.
• Parasite occurrence is common in adult opossums.

## Graphical abstract

**Figure:**
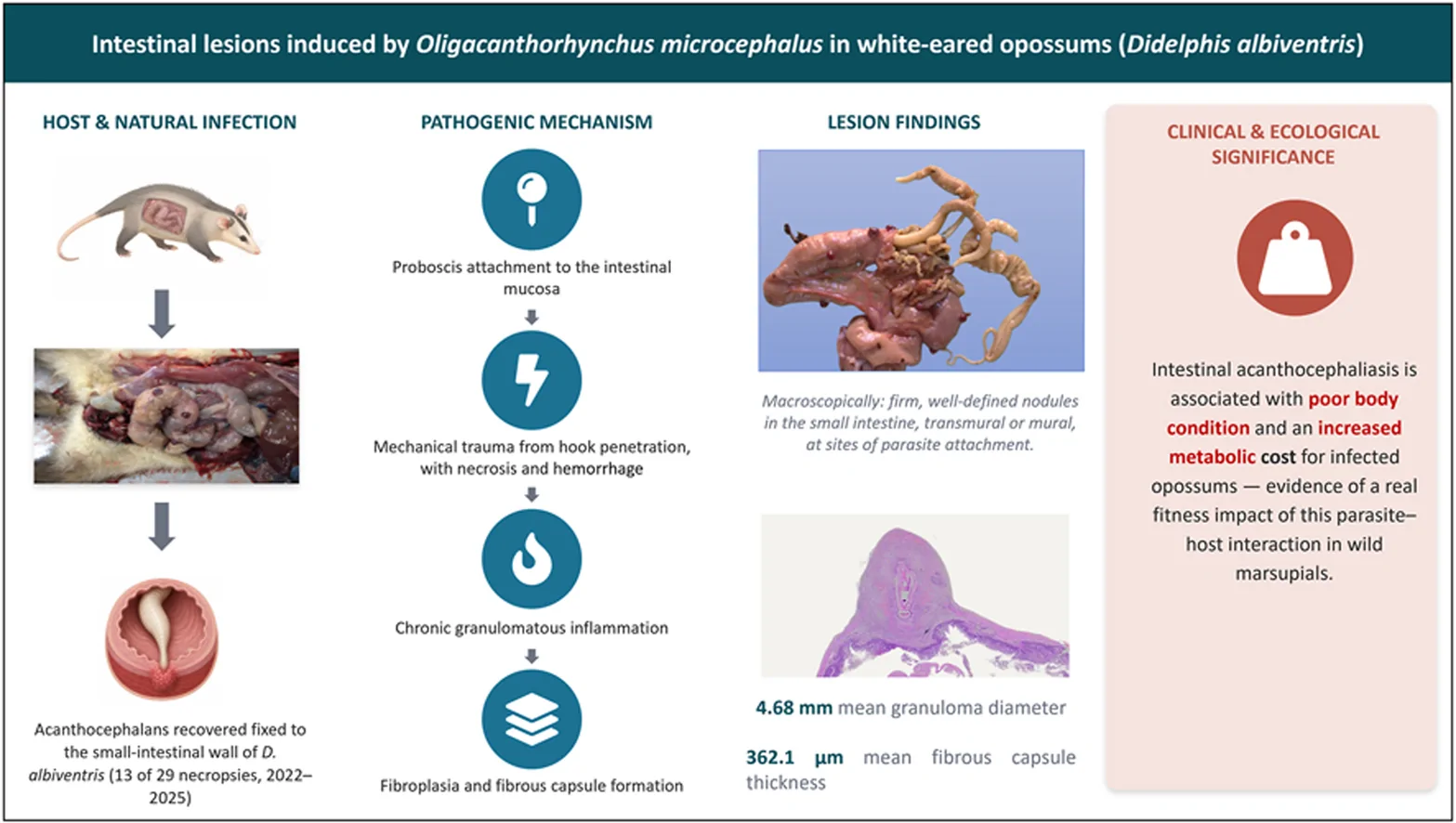

## 1 Introduction

Opossums are marsupials widely distributed across South America and are frequently found in urban and peri-urban environments due to their ecological plasticity and opportunistic behavior. As omnivores, they eat fruits, insects, and small vertebrates (Bezerra-Santos et al., 2021). White-eared opossums (*Didelphis albiventris*), notable for their synanthropy, are often seen in urban parks, gardens, vacant lots, peridomestic areas, and near homes and domestic animal dwellings (Bezerra-Santos et al., 2021; Biolchi et al., 2021). This close contact with anthropized environments exposes them to toxic, infectious, and parasitic agents, including gastrointestinal helminths (Oliveira Neto et al., 2022; Bezerra-Santos et al., 2021; Biolchi et al., 2021).

Acanthocephalans exhibit a heteroxenous life cycle, using arthropods as intermediate hosts and various vertebrates fish, amphibians, reptiles, birds, and mammals as definitive hosts for their adult form (Richardson, 2006; Sgroi et al., 2024). There are also uncommon occurrences of human acanthocephaliasis (Lotfy, 2020). These helminths attach to the host's intestinal wall with a retractable, hooked proboscis, penetrating the tissue and causing focal necrosis, hemorrhage, and a tissue inflammatory response (George and Nadakal, 1981; Richardson and Barnawell, 1995; Irshadullah and Mustafa, 2012; La Sala et al., 2013; Bazh and Hamouda, 2021; Caballero-Viñas and Sánchez-Nava, 2020; Sgroi et al., 2024). The resulting nodular lesions, characterized as granulomas, are composed of epithelioid macrophages, multinucleated giant cells, lymphocytes, and encapsulating connective tissue (George and Nadakal, 1981; Richardson and Barnawell, 1995; Gonzales-Viera et al., 2009; Sgroi et al., 2024).

Intestinal acanthocephaliasis is reported in various vertebrates (Richardson and Barnawell, 1995; Gonzales-Viera et al., 2009; Sgroi et al., 2024). However, in neotropical marsupials, especially *D. albiventris,* morphological studies of these lesions are limited, as most research focuses on parasite diversity (Souza et al., 2017; Bezerra-Santos et al., 2021), and descriptions of tissue responses are scarce.

Previous studies on acanthocephalan infections in opossums focused mainly on chronic fibrotic lesions in North American species (Richardson and Barnawell, 1995), while intestinal pathology in fish, blue-winged teal and wild boar (George and Nadakal, 1981; Richardson and Barnawell, 1995; Irshadullah and Mustafa, 2012; Caballero-Viñas and Sánchez-Nava, 2020; Bazh and Hamouda, 2021; Sgroi et al., 2024) has also been described (Sgroi et al., 2024). However, a detailed pathological and morphometric characterization of intestinal lesions specifically caused by *O. microcephalus* in *D. albiventris* has not been documented. The present study addresses this gap by analyzing a larger series of cases and describing the dynamic progression of lesions, ranging from acute necrosis and active inflammation at the site of proboscis penetration to organized granulomas with fibrocollagenous remodeling, neovascularization, and occasional mineralization. This detailed characterization, supported by morphometric data, expands comparative knowledge of acanthocephalan pathology in neotropical marsupials and advances beyond the chronic scarring lesions previously reported. In this context, characterizing these lesions is relevant for understanding parasite–host interactions and elucidating the inflammatory patterns induced by *O. microcephalus* infection in free-living white-eared opossums (D.*albiventris*).

## 2 Materials and methods

### 2.1 Ethical aspects

The project that includes this study was submitted to the Ethics Committee on the Use of Animals (CEUA) and was declared exempt from analysis, as it did not involve the use of live vertebrate animals, in accordance with Law No. 11,794, of October 8, 2008, of Brazil.

### 2.2 Animal selection and material collection

This study is based on a retrospective survey of necropsies of free-living white-eared opossums (*D. albiventris*) recorded at the Veterinary Pathology Laboratory, Veterinary Hospital, Federal University of Mato Grosso (UFMT), Cuiabá, Brazil, from December 2022 to April 2025. During the study, 29 individuals were necropsied, and 13 cases with acanthocephalans in the intestinal lumen (Cases 1-13) were selected. Animals with visceral liquefaction or missing abdominal viscera due to predation or *post-mortem* trauma were excluded. Additional biological information was collected from referral documents and, when available, clinical records. Age was estimated by body length, weight, and dentition (Gentile et al., 1995). Body condition was rated on a scale of 1 to 5 (1 = lean, 5 = obese). Data on parasite burden and intestinal alterations were extracted from necropsy reports, In these records, the gastrointestinal tract had removed in its entirety (*en bloc*, i.e., as a single piece) and examined for mucosal integrity, luminal distension, wall thickening or thinning, and nodules. The entire intestine was opened longitudinally along the mesenteric border, and its contents were examined for helminths. Specimens from the lumen or attached to intestinal nodules were manually removed or retrieved with forceps, then sent to parasitology labs for taxonomic identification.

Parasite burden was categorized semi-quantitatively, including both adult and immature specimens, according to the number of parasites observed in the intestinal lumen and their association with macroscopic lesions. Low burden was defined as ≤5 parasites with minimal or no luminal dilation; medium burden as 6–15 parasites with partial lumen filling and moderate nodular involvement; and high burden as >15 parasites with extensive lumen filling, multiple nodules, and frequent wall thinning.

Recovered helminths were washed in physiological solution and preserved in 70% ethanol. For morphological analysis, helminths were cleared in lactophenol or 90% phenol (depending on specimen quality and thickness) and examined under light microscopy. When needed, specimens were stained with Langeron's carmine to improve visualization of diagnostic structures, as described by Hoffmann (1987). Identification was based on proboscis morphology, including the number and arrangement of hooks and overall parasite form, following taxonomic criteria for Acanthocephala (Schmidt, 1972; Freitas et al., 2022). Images of acanthocaphalans were captured using a Nikon SMZ 745T stereomicroscope with an attached PrimeCam 12 Pro camera and a Nikon Eclipse SI RS light microscope with an attached PrimeCam Intervision NPlus 12 camera. As the camera attached to the stereomicroscope did not provide precise scale data, the scale bar was added after the image was acquired.

Acanthocephalan parasite load was assessed semi-quantitatively, including both adult and immature specimens, based on intestinal lumen occupation, wall changes, and mural nodules. Lesion severity was categorized by the authors according to a combination of parasite burden, macroscopic findings, and histological lesion severity: mild (few parasites, no distension, minimal histological changes), moderate (partial lumen filling, slight wall alteration, limited histological lesions), or severe (extensive lumen filling, marked distension, multiple nodules, and pronounced histopathological changes). This classification was proposed by the authors specifically for this study, as no published reference was available.

### 2.3 Histopathological examination

Intestinal fragments containing nodules were collected and fixed in 10% buffered formalin. After fixation, the tissues were routinely processed for histological slide preparation and stained with hematoxylin and eosin (H&E) and Masson's trichrome (Prophet et al., 1995).

Histological analysis considered the architecture of the intestinal wall layers, the composition of the inflammatory infiltrate, the degree of tissue destruction, the arrangement of fibrovascular proliferation, and the presence of parasitic structures. Lesions were classified semi-quantitatively according to type, distribution (focal, multifocal, or diffuse), and intensity, graded as absent (−), mild (+), moderate (++), or severe (+++) (Erben et al., 2014; Barros and Rissi, 2021)

To ensure methodological consistency, findings were further categorized as “adequate,” “limited,” or “not assessable.” This classification was based on tissue preservation, completeness of the section, and the presence of artifacts that could compromise interpretation. Inflammatory infiltrates and fibrosis were scored semi-quantitatively, where “+” indicated mild changes, “++” moderate changes, and “+++” severe changes. These categories were adapted from previously published semi-quantitative systems in veterinary pathology (Erben et al., 2014; Barros and Rissi, 2021), with minor adjustments to fit the specific lesions observed in *D*. *albiventris*.

All slides were independently examined by two experienced pathologists. In cases of discordant interpretation, a consensus was reached after joint review. Inter-observer agreement was qualitatively assessed to ensure consistency in scoring. This standardized approach was adopted to increase reproducibility and transparency of the histopathological analysis.

### 2.4 Intestinal nodules morphometry

Intestinal nodules morphometry was performed on nine animals (Cases 1, 2, 5, 6, 9-13), with the others excluded due to marked autolysis or inappropriate histological sectioning. Sections stained with Masson's trichrome were used to facilitate delineation of structural zones (Richardson and Barnawell, 1995). The histological sections were evaluated using a Leica Ivesta 3 Greenough stereomicroscope (Leica Microsystems, Wetzlar, Germany), equipped with a scale calibrated for morphometric measurements. The measurements were performed directly on histological slides and reported in micrometers (μm) or millimeters (mm), depending on the size of the structure evaluated. Whole, well-defined granulomas in the cutting plane were included with up to three representative granulomas per animal.

The morphological variables measured comprised the total granuloma diameter, thickness of the peripheral collagenized capsule, thickness of the cellular granulomatous zone, and thickness of the submucosa underlying the granuloma (Richardson and Barnawell, 1995). The granuloma diameter was determined as the average of the lesion's largest and perpendicular axes, an approach compatible with morphometric analyses of granulomatous structures (Amaral et al., 2017).

Capsule thickness was measured at three to four radial sections distributed along the circumference of the fibrovascular tissue, from the outer limit of the collagen deposition to the transition with the cellular granulomatous zone, in accordance with histological descriptions of perilesional fibrous organization (Richardson and Barnawell, 1995). The cellular inflammatory zone was measured by radial measurements extending from the center of the cellular margin to the beginning of the peripheral collagenized capsule. Submucosal thickness was determined by the distance between the base of the muscularis mucosae and the muscular layer immediately adjacent to the lesion, and compared with respective regions of submucosa considered histologically preserved in the same section, considering that chronic inflammatory processes can promote significant submucosal thickening (Richardson and Barnawell, 1995). When parasitic structures were present in the lesions, two perpendicular measurements (major and minor axes) were taken, and the arithmetic mean of the measurements was subsequently calculated. For each morphometric variable, the arithmetic mean and standard deviation were calculated. In cases where two or more granulomas were present in the same animal, up to three representative granulomas were selected. Initially, the individual means of each granuloma were calculated. Subsequently, the mean of the granulomas evaluated in each animal was calculated, yielding a single representative value per individual, which was considered the final sampling unit, as recommended for the standardization of quantitative analyses in histopathological studies (Amaral et al., 2017).

## 3 Results

### 3.1 Study animals

During the study period, 29 opossums (*D. albiventris*) were evaluated, with 19/29 (65.5%) adults, 8/29 (27.6%) pups, and 2/29 (6.9%) juveniles. Among the 19 adult individuals, 13 (68.5%) (Table 1) presented intestinal parasitism by acanthocephalans, associated with intestinal lesions characterized by one or multiple nodules in the intestinal wall, corresponding to the attachment sites of the parasites. All specimens included in this study originated from the surroundings of parks, squares, or wooded areas in the urban area of the municipality of Cuiabá, Mato Grosso, Brazil. Body weight was recorded in 12/13 (92.3%) of the animals, ranging from 570 to 1615 g, with a mean of 1217.5 g and a median of 1297.5 g. Body condition was classified on a scale of 1 to 5 (1 = lean, 5 = obese), with the majority (9/13; 69.2%) classified as lean and 4 as normal. Intestinal histological analysis status was considered adequate in most cases (8/13; 61.5%), followed by limited (3/13; 23.1%) and not assessable (2/13; 15.4%).

**Table 1:** Information on individuals case, body condition score, the severity of intestinal parasitism, the main findings associated with *Oligacanthorhynchus microcephalus* infection, and the primary causes of death in white-eared opossums (*Didelphis albiventris*) was analyzed.

Case	Sex	Score	Estimated acanthocephalan burden	Nodules	Intestinal lumen dilation	Primary cause of death
1	M	1	High	22	+++	Multisystemic inflammatory disease
2	M	2	High	10	+++	Multisystemic inflammatory disease (Euthanasia Ex Extremis)
3	F	2	medium	3	++	Trauma (predation)
4	M	2	Low	1	+	Trauma (predation)
5	M	2	High	3	+	Trauma (high-energy impact)
6	F	3	medium	2	-	Trauma (high-energy impact)
7	M	2	medium	8	+	Trauma (predation)
8	M	2	Low	1	+	Trauma (high-energy impact)
9	M	3	medium	2	-	Trauma (high-energy impact)
10	M	2	medium	4	+	Trauma/bacterial sepsis (secondary to predation) and parasitic granuloma in mesentery/peritonitis
11	M	2	medium	7	+	Multisystemic inflammatory disease
12	F	3	High	7	++	Severe trauma (high-energy impact) and parasitic granuloma in the omentum
13	F	2	High	8	+	Trauma (predation) (Euthanasia Ex Extremis)

Notes: M = male; F = female; Score 1–5 (1 = cachexia; 5 = obesity); NI = not informed; Luminal dilation: absent (−), mild (+), moderate (++), severe (+++).

### 3.2 Morphological characterization of *Oligacanthorhynchus microcephalus*

**Fig. 1:**
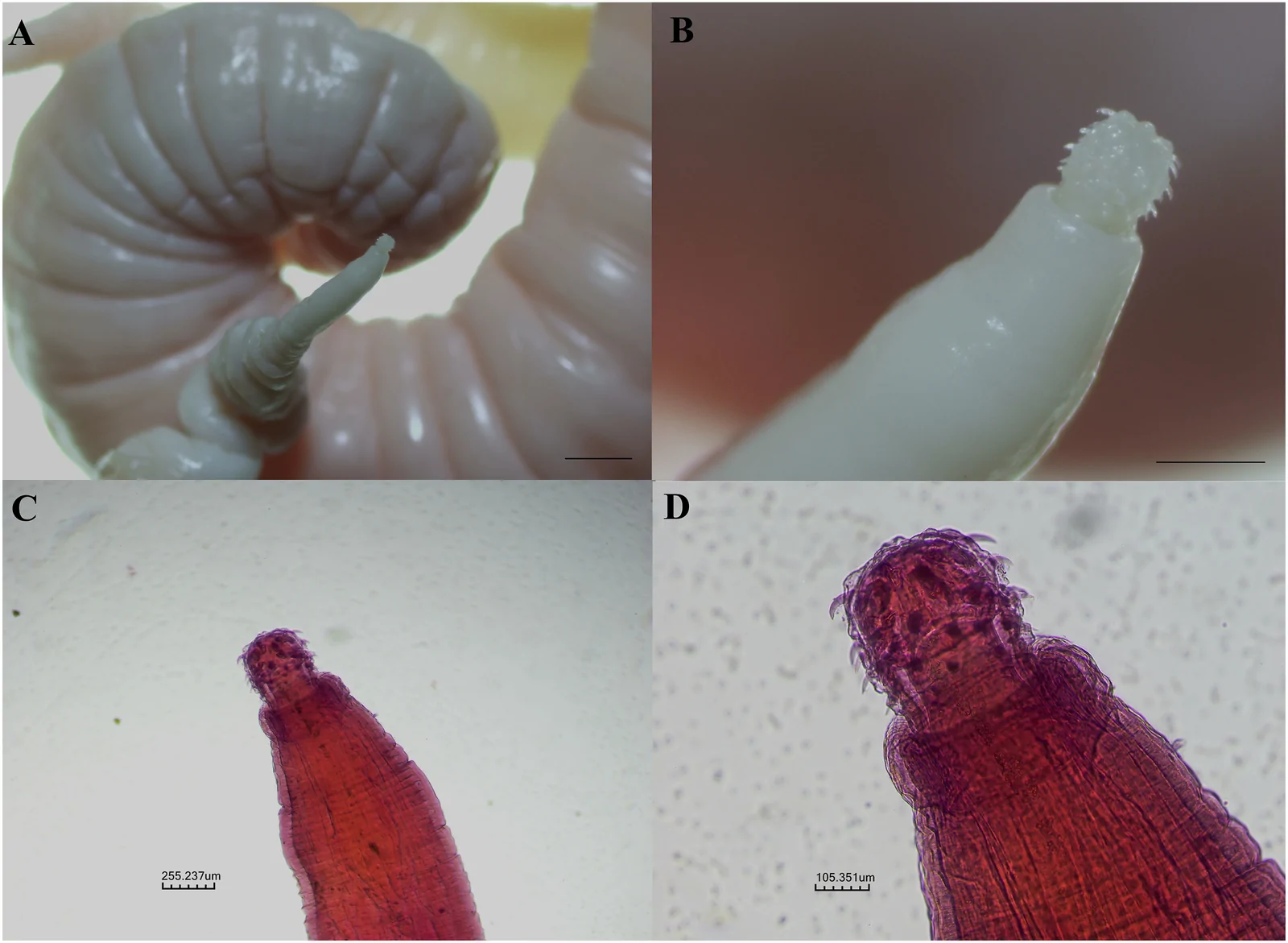
Light micrograph of adult *Oligacanthorhynchus microcephalus* associated with intestinal granulomas in white-eared opossums (*Didelphis albiventris*) naturally infected. A. General view of the segmented body, showing the more tapered anterior end and evidencing the proboscis. Scale bar = 2000 μm. B. Detail of the proboscis apex, armed with spiral rows of hooks. Scale bar = 450 μm. C–D. Anterior region at higher magnification, stained with acid carmine and dehydrated through a graded alcohol series, highlighting the proboscis and its hooks.

The parasites were identified as *Oligacanthorhynchus microcephalus* (Fig. 1). Five male and five female *O. microcephalus* were measured. Males body length 64.72 mm ± 3.83 mm (59-69 mm); maximum width 3.9 ± 0.5 (3.5–4.8) mm; proboscis length 246.6 ± 9.1 (238–260) μm; and proboscis width 232.4 ± 3.9 (229–238) μm. Females measured: body length 160.0 ± 29.1 (120–192) mm; maximum width 8.5 ± 0.8 (7.4–9.5) mm; Proboscis length 267.8 ± 15.0 (254–289) μm; and proboscis width 288.4 ± 19.2 (258–310) μm. The specimens were deposited in the Helminthological Collection of the Federal University of Jataí under the codes CHUFJ-0084, CHUFJ-0085, and CHUFJ-0086.

### 3.3 Macroscopic pathological findings

All parasitized opossums (13/13; 100%) presented intestinal lesions characterized by multiple nodules in the intestinal wall that corresponded to the parasitic attachment sites. Animal data, macroscopic alterations, and cause of death are summarized in Table 1.

**Fig. 2:**
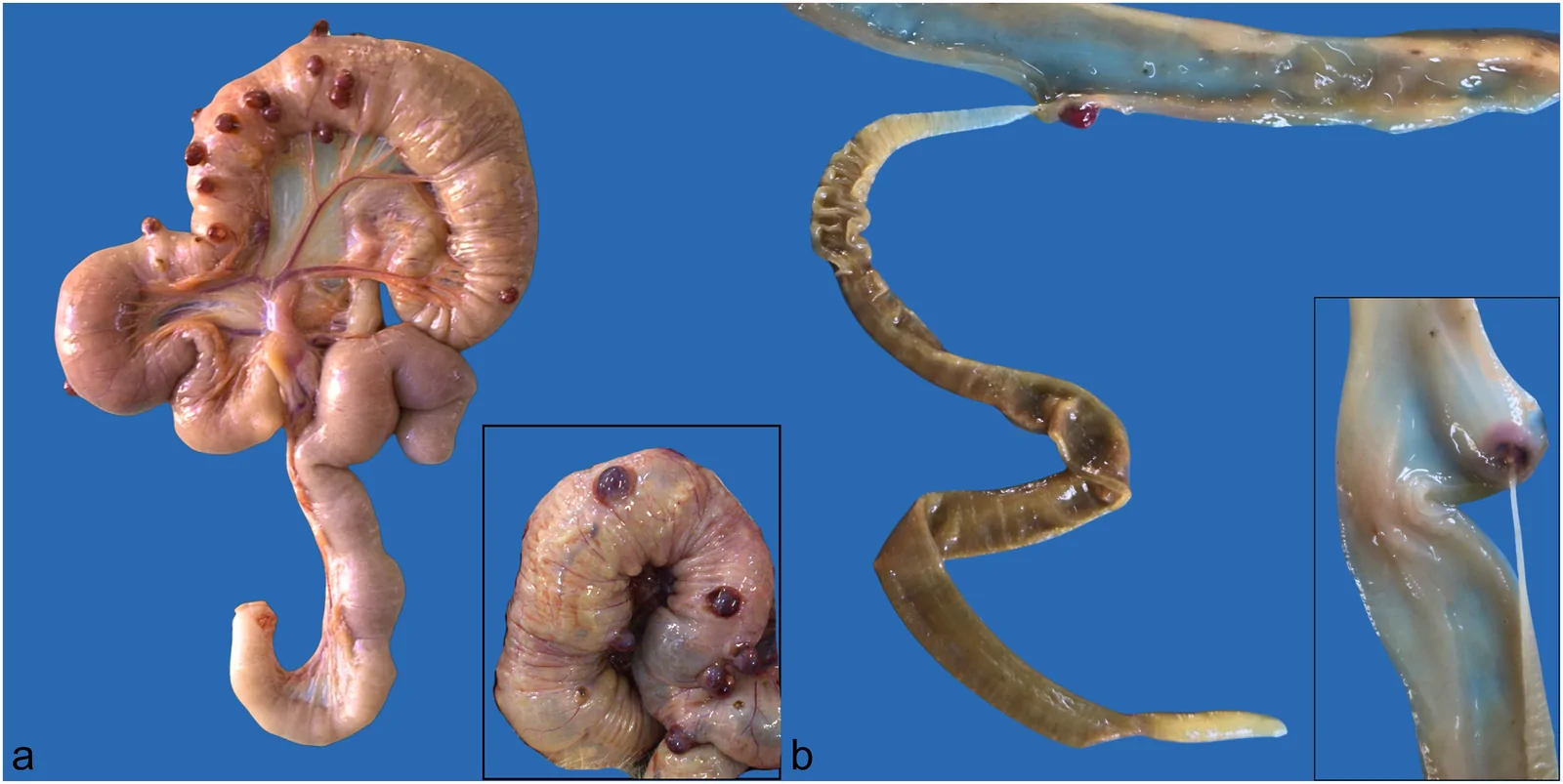
Intestinal lesions induced by *Oligacanthorhynchus microcephalus* in white-eared opossums (*Didelphis albiventris*). a) Dilated small intestine, with thinned wall and containing multiple exophytic red nodules. Inset; close-up view of panel, highlighting the protruding intestinal nodules on the serosal surface. Case 1. b) Note the entire parasite attached to the intestinal nodule. Inset: detail of the attachment site of the acanthocephalan proboscis with firm adherence and local redness. Case 10.

Macroscopically, the serosa of the small intestine of all opossums evaluated (13/13; 100%), predominantly in the jejunum, presented mural nodules. The nodules were firm, rounded, well-defined, and varied in color from pale yellow to intense red, measuring 1-10 mm in diameter. The number of nodules per individual ranged from 1 to 22 (average: 5 per animal). Luminal dilation was observed in 11/13 animals (85%), associated with the presence of intraluminal acanthocephalans, attached or not to the mucosa and/or locations corresponding to the nodules (Fig. 2a and b). In four of these cases, thinning of the intestinal wall due to luminal repletion was observed, allowing visualization of the parasites through the wall. In Case 12, there was a nodule in the omentum that surrounded and adhered to one of the intestinal nodules. In Case 10, peritonitis was caused by a mesenteric nodule that adhered to the intestinal wall. Based on the methodological criteria, parasite load was classified as high in 5/13 (38.5%), medium in 6/13 (46%), and low in 2/13 (15.5%) of the animals.

### 3.4 Microscopic findings

**Fig. 3:**
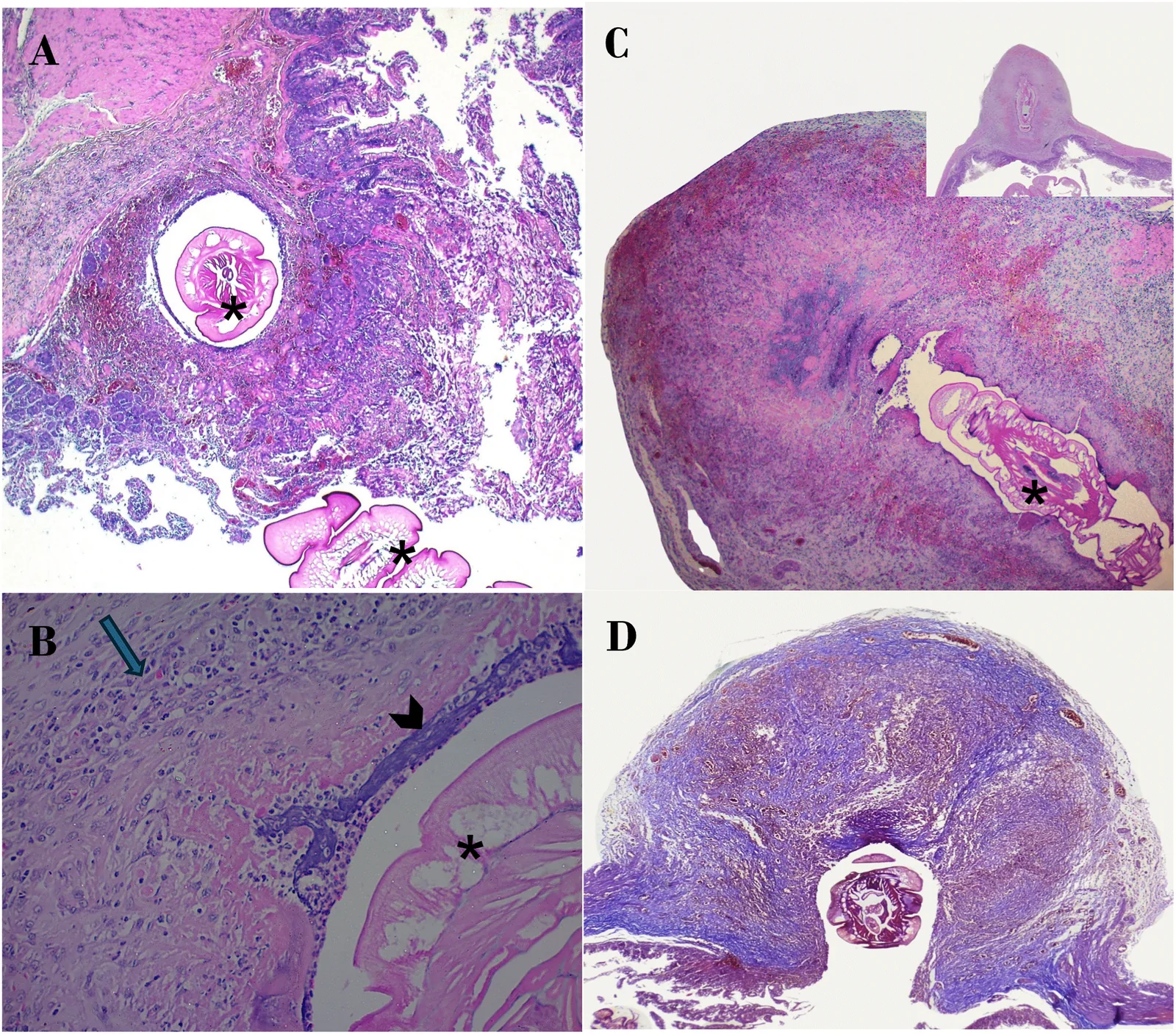
Intestinal lesions induced by *Oligacanthorhynchus microcephalus* in white-eared opossums (*Didelphis albiventris*). Photomicrographs of intestinal tissue: **A)** Overview of the intestine showing parasite attachment with marked mucosal obliteration, necrosis, hemorrhage, and acute inflammation surrounding the structure of *O. microcephalus* (*). Case 1. H&E, Obj. 4×. **B)** Section of inflammatory granuloma containing parasite (*), surrounded by a thin irregular layer of cellular debris and granulomatous inflammation (arrowheads), with dense fibroblast cellularity. Case 18. H&E, Obj. 20×. **C)** Cross-section of the proboscis (*) centrally located within the nodule, surrounded by fibrocollagenous tissue, intense neovascularization, and mild mononuclear infiltration. Case 1. H&E, Obj. 10×. Inset: Scar nodule with fibrocollagen deposition, neovascularization, and reduced inflammatory cell density extending from submucosa to serosa, showing mucosal reorganization. Case 27. H.E. Obj. 2.5×. **D)** Histological section stained with Masson's trichrome, highlighting organized fibrovascular nodules in chronic lesions. Case 1. Obj. 10×.

Histological analysis was possible in 11/13 cases (84.6%). Post-mortem artifacts limited mucosal assessment in four animals. Nodules varied between granulomas with viable parasites and fibrotic granulomas with collagen deposition and neovascularization. Most were transmural, with necrosis, hemorrhage, and mixed inflammatory infiltrates (Table 2, Fig. 3a–d).

**Table 2:** Histopathological evaluation of intestinal nodules associated with *Oligacanthorhynchus microcephalus* infection in free-living white-eared opossums (*Didelphis albiventris*), including lesion extent, inflammatory component, fibrosis, neovascularization, and distribution of inflammatory cell populations.

Case	Status of intestinal hitological analysis	Nodule extension	Inflammatory infiltrate					Fibrosis	Neovascularization
			Neutrophils	Lymphocytes	Plasma cells	Macrophages	Eosinophils		
1	Adequate	T	++	+	+	+++	+	++	+++
2	Adequate	T	+++	++	+	+++	+	++	++
3	Limited	T	+	+	-	++	+	+++	++
4	not assessable	N/E	N/E	N/E	N/E	N/E	N/E	N/E	N/E
5	Adequate	SM-S	++	+	-	+++	-	++	++
6	Limited	T	-	++	-	+++	+	++	++
7	Limited	T	+++	++	+	++	-	+++	++
8	not assessable	N/E	N/E	N/E	N/E	N/E	N/E	N/E	N/E
9	Adequate	SM-S	+++	+++	-	+++	+	++	++
10	Adequate	T	+	+++	++	+++	+	+++	+++
11	Adequate	SM-S	+++	++	++	+++	-	+++	+++
12	Adequate	T	+++	+++	+	+++	+	+++	++
13	Adequate	T	+++	+++	++	++	++	+++	++

Notes: T: transmural; SM-S: submucosal to serosa; N/E: not evaluated due to autolysis.

### 3.5 Morphometry of intestinal nodules

The diameter of the nodules ranged from 3.2 to 11.13 mm (mean 4.68 ± 2.65 mm). The thickness of the collagenized capsule ranged from 161.9 to 607.6 μm (mean 362.1 ± 165.8 μm). At the same time, the granulomatous zone with a mixed cellular infiltrate, fibroplasia, neovascularization, and an epithelioid component ranged from 1.28 to 4.7 mm (mean 1.99 ± 1.14 mm). The submucosal layer adjacent to the granuloma presented a thickness between 137 and 350.5 μm (mean of 279.1 ± 63.9 μm), compared to the normal submucosa, which ranged from 66.9 to 132.3 μm (mean of 105.4 ± 21.9 μm). The diameter of the parasite attachment structure, measured in three cases inside the nodules, ranged from 650 to 978.8 μm (mean of 785.9 ± 171.6 μm).

## 4 Discussion

The results of this study demonstrate that *O. microcephalus* infection in *D. albiventris* is related to the formation of mural or transmural intestinal nodules resulting from persistent parasitic attachment to the intestinal wall. These lesions reflect a granulation process triggered by mechanical trauma at the site of proboscis and hook attachment and may be associated with varying degrees of granulomatous inflammation. The consistent spatial association between the nodules observed macroscopically and the parasitic attachment sites identified histologically supports the central role of the parasite in the pathogenesis of these lesions (George and Nadakal, 1981; Richardson and Barnawell, 1995; Gonzales-Viera et al., 2009; Irshadullah and Mustafa, 2012; La Sala et al., 2013; Lotfy, 2020; Richardson et al., 2014; Bazh and Hamouda, 2021). Despite the limitations imposed by study duration and sample size, the study reflects natural data and illustrates the behavior of parasitic infection and its host–parasite relationship. External factors could not be measured and were therefore not statistically compared, as this would require a case–control design, which is difficult to implement in wild animals, particularly when considering the principles of reduction, refinement, and replacement in animal research.

Although *O. microcephalus* has already been reported as part of the intestinal parasitic fauna of *D. albiventris* in different regions of Brazil (Souza et al., 2017; Bezerra-Santos et al., 2021), and from other host species in different countries, the anatomopathological changes associated with infection in *D. albiventris* remain poorly documented. The present study provides novel contributions by characterizing the morphological patterns of intestinal lesions, detailing the inflammatory response induced by acanthocephalans, and highlighting the association with poor body condition in free-living opossums from urban environments. These findings provide novel insights into parasite-host interactions in neotropical marsupials and add a pathological perspective not addressed in previous reports. Moreover, chronic helminth infections are known to modulate host immune responsiveness and may influence susceptibility to other pathogens (Borkow et al., 2000; Mabbott, 2018), highlighting the broader implications of the inflammatory responses observed in *D. albiventris*.

In four animals, the observed luminal repletion caused the shortening of the villi. In addition, in multiple sections of the parasitic attachment nodules analyzed, deep insertion of the proboscis into the mucosa and submucosa was observed, causing architectural disarray, focal destruction of the villi, necrosis, local hemorrhage, and active inflammation adjacent to the nodules. These alterations are consistent with findings in vertebrate hosts, in which parasitic attachment results in significant structural changes in the intestinal mucosa, including epithelial abrasion and loss of villi (de Buron and Nickol, 1994; Irshadullah and Mustafa, 2012; Matos et al., 2017), as well as focal necrosis, hemorrhage, and inflammation of the intestinal wall layers (George and Nadakal, 1981; Richardson and Barnawell, 1995; Gonzales-Viera et al., 2009; Irshadullah and Mustafa, 2012; La Sala et al., 2013; Richardson et al., 2014; Bazh and Hamouda, 2021).

Morphometric analysis provides quantitative evidence that these nodules do not merely represent localized inflammatory foci but expansive structures associated with intestinal wall remodeling. In chronic cases, the fibrocollagenous component predominated over the inflammatory infiltrate, forming scar nodules that partially or completely replaced the layers of the intestinal wall. This progressive organization is characterized by tissue expansion, fibrous proliferation, neovascularization, and increasing collagen deposition in the lesion (George and Nadakal, 1981; Amin, 1985; Gonzales-Viera et al., 2009; Irshadullah and Mustafa, 2012; Caballero-Viñas and Sánchez-Nava, 2020). Most lesions showed transmural extension, involving multiple layers of the intestinal wall, consistent with the expected progression when aggressive proboscis attachment is present, surpassing the mucosa and reaching the submucosa, muscularis, and serosa (Caballero-Viñas and Sánchez-Nava, 2020). Despite frequent transmural involvement, lesions remained confined to the intestinal wall in most cases, except in two, where granulomas were found in the omentum and mesentery, likely related to accidental perforation of the intestinal wall. Similar lesions associated with acanthocephalans have been described in other vertebrate hosts, including birds and mammals (Gonzales-Viera et al., 2009; La Sala et al., 2013) and in opossums (Richardson and Barnawell, 1995), parasitized by *O. tortuosa* (currently recognized as a synonym of *O. microcephalus*) (Richardson et al., 2014).

Granulomatous inflammation at the site of attachment of *O. microcephalus* showed a predominance of epithelioid macrophages, few giant cells, necrosis, and dystrophic mineralization. This intense response occurs predominantly around sections of the proboscis of structurally intact acanthocephalans, emphasizing that the host response acts mainly in the local containment of mechanical damage (Richardson and Barnawell, 1995; Gonzales-Viera et al., 2009; Richardson et al., 2014; Chen et al., 2018; Sgroi et al., 2024), not proving competent to eliminate the helminth (Sgroi et al., 2024). Taken together, the results indicate that *O. microcephalus* infection in *D. albiventris* is associated with a pathogenetic sequence that begins with mechanical trauma at the proboscis attachment site, evolves into local granulomatous inflammation, and progresses to fibrovascular and fibrocollagenous remodeling of the intestinal wall (Richardson and Barnawell, 1995). From a morphological perspective, the intestinal nodules observed do not correspond exclusively to classic granulomas, but rather to different stages of a mural remodeling response associated with parasitic attachment.

External factors and environmental events may influence the impact of parasites on wild animal populations, although these were not assessed in the present study, although these were not assessed in the present study. Intestinal lesions associated with acanthocephalan infection were frequently extensive, however, intestinal acanthocephaliasis could not be established as the primary cause of death in the opossums evaluated. Of the 13 individuals, in 10 the immediate cause of death was associated with high-energy trauma or predation, while three individuals presented severe clinical deterioration and death prior to emergency care or were subjected to humane euthanasia (Bianchi-Morais et al., 2026). No direct relationship could be established between compromised clinical conditions due to intestinal acanthocephaliasis and greater susceptibility to predation or collisions. Poor body condition often coincided with compromised intestinal mucosal integrity and parasite load (Richardson and Barnawell, 1995; Caballero-Viñas and Sánchez-Nava, 2020), however, this observation was not supported by statistical analyses, and the potential contribution of other parasitic infections to poor body condition was not assessed. Altered intestinal architecture, coupled with the metabolic cost of persistent inflammation, can contribute to progressive skill deficits, especially in free-living animals exposed to environmental stressors (Borkow et al., 2000; Mabbott, 2018; Megha et al., 2021; Xing et al., 2024), but this remains a hypothesis requiring further investigation.

The scarcity of information prior to deaths limited an accurate assessment of the apparent increase in necropsies performed between 2022 and 2025. The frequency of *O. microcephalus* observed in opossums necropsied during the study period was high (44.8%), rising to 68.4% when only adult *D. albiventris* were considered. Population change patterns related to the pandemic (Sgroi et al., 2024) or to climatic incidents (Short et al., 2017; Cohen et al., 2020), such as the large fires in cerrado and pantanal areas surrounding the site of this study, in the Metropolitan Region of Cuiabá, MT, Brazil, were not evaluated in either the opossum population or potential invertebrate hosts of acanthocephalans. Nevertheless, the frequency observed in this study is compatible with previous data in didelphids, in which *O. microcephalus* was identified in 69.2% of adult *D. marsupialis* in northern Mato Grosso (Freitas et al., 2022).

Parasites and wild hosts often coexist without evident clinical signs, including their relationship with acanthocephalans. It is well known that external factors or environmental changes may modify the way parasites affect wild animal populations. Although such influences could not be measured in the present sampling, they should be considered an important factor in the host–parasite relationship. Despite the recognized resilience of *D*. *albiventris*, our study demonstrates significant intestinal morphological alterations associated with infection by *O. microcephalus* in this species. These findings underscore the importance of integrating pathological, ecological, and environmental perspectives to advance the understanding of host–parasite dynamics in neotropical marsupials.

## CRediT authorship contribution statement

**Julieni Bianchi-Morais:** Writing – original draft, Visualization, Validation, Investigation, Formal analysis, Conceptualization. **Luis Jhordy Alfaro Quillas:** Visualization, Investigation, Formal analysis. **Wuglenya Daislla Martins da Silva:** Visualization, Supervision, Methodology, Data curation. **Victoria Luiza de Barros Silva:** Supervision, Investigation, Conceptualization. **Dirceu Guilherme de Souza Ramos:** Writing – review & editing, Visualization, Methodology, Formal analysis, Data curation, Conceptualization. **Richard de Campos Pacheco:** Writing – review & editing, Validation, Methodology, Investigation, Formal analysis, Data curation. **Fernando Henrique Furlan:** Writing – original draft, Supervision, Methodology, Investigation. **Nadia Alini Bobbi Antoniassi:** Writing – review & editing, Supervision, Methodology, Formal analysis, Conceptualization. **e Edson Moleta Colodel:** Writing – review & editing, Methodology, Investigation, Funding acquisition, Data curation, Conceptualization.

## Funding

This study was funded by a master's scholarship from the Coordination for the Improvement of Higher Education Personnel - Brazil (CAPES) and by The Mato Grosso State Secretariat for the Environment (SEMA-MT) provided logistical and financial support (Agreement Term No. 2607/2025/SEMA/MT, SIGADOC Process: SEMA-PRO-2024/11198).

## Declaration of competing interest

The authors declare the following financial interests/personal relationships which may be considered as potential competing interests: Julieni Bianchi-Morais reports financial support was provided by Coordination for the Improvement of Higher Education Personnel (CAPES) - Brazil. Edson Moleta Colodel reports financial support and administrative support were provided by Mato Grosso State Secretariat for the Environment (SEMA-MT) - Brazil. If there are other authors, they declare that they have no known competing financial interests or personal relationships that could have appeared to influence the work reported in this paper.
